# Pathogenesis of Chronic Urticaria: An Overview

**DOI:** 10.1155/2014/674709

**Published:** 2014-07-10

**Authors:** Sanjiv Jain

**Affiliations:** Skin Care Clinic, 108 Darya Ganj, New Delhi 110002, India

## Abstract

The pathogenesis of chronic urticaria is not well delineated and the treatment is palliative as it is not tied to the pathomechanism. The centrality of mast cells and their inappropriate activation and degranulation as the key pathophysiological event are well established. The triggering stimuli and the complexity of effector mechanisms remain speculative. Autoimmune origin of chronic urticaria, albeit controversial, is well documented. Numerical and behavioral alterations in basophils accompanied by changes in signaling molecule expression and function as well as aberrant activation of extrinsic pathway of coagulation are other alternative hypotheses. It is also probable that mast cells are involved in the pathogenesis through mechanisms that extend beyond high affinity IgE receptor stimulation. An increasing recognition of chronic urticaria as an immune mediated inflammatory disorder related to altered cytokine-chemokine network consequent to immune dysregulation resulting from disturbed innate immunity is emerging as yet another pathogenic explanation. It is likely that these different pathomechanisms are interlinked rather than independent cascades, acting either synergistically or sequentially to produce clinical expression of chronic urticaria. Insights into the complexities of pathogenesis may provide an impetus to develop safer, efficacious, and targeted immunomodulators and biological treatment for severe, refractory chronic urticaria.

## 1. Introduction

Chronic urticaria is a distressing disorder that adversely impacts the quality of life; yet its pathogenesis is not well delineated and, accordingly, the treatment is often palliative and therapeutic outcome is suboptimal. This necessitates an understanding of the pathogenesis to facilitate development of improved therapies. In the recent past rapid strides in understanding the pathomechanism of chronic urticaria have been recorded; yet, most of the evidence based, seemingly impregnable and conclusive hypotheses have been countered by alternative, equally authentic, convincing and logistic counter explanations.

The knowledge of molecular immunopathogenesis and complexities of effector mechanisms in chronic urticaria has been enhanced by immunohistologic studies performed on sequential biopsies of urticarial wheals and focused on infiltrating cell immunophenotypes and related cytokines, chemokines/chemokine receptors, and adhesion molecules [1].

The urticarial wheal is characterized by dermal edema, vasodilatation, and perivascular nonnecrotizing infiltrate comprising primarily of mononuclear cells, predominantly CD4+ lymphocytes, with variable numbers of monocytes, neutrophils, eosinophils, and basophils [2–4]. The dermal neutrophilia is strikingly evident at sixty minutes of evolution of wheal with neutrophils representing the main component of the cellular infiltrate [4]. Mast cell numbers remain unaltered and are comparable to those in uninvolved skin and healthy controls [4, 5]. The cytokine profile is characterized by an increase in interleukin-4 (IL-4), interleukin-5 (IL-5), and interferon-gamma RNA (IFN-gamma), suggestive of a mixed Th 1/Th 2 response. Chemokines are upregulated and increased expression of adhesion molecules is evident. The uninvolved skin is characterized by upregulation of soluble mediators and adhesion molecules, almost identical to lesional skin, and significantly higher T-cell numbers, while accumulation of neutrophils is an exclusivity of whealing skin [4] (Tables 1 and 2).

Chronic urticaria is initiated by inappropriate activation and degranulation of dermal mast cells. This key pathophysiological event is predominant at the very onset and the released cellular contents prime the immediate phase of inflammation, which progresses to a complex interplay of varied proinflammatory mediators, cytokines, chemokines, chemokine receptors, and adhesion molecules that regulate vasoactivity and specific kinetics of cellular infiltration, ultimately evolving into a lymphocyte and granulocyte mediated hypersensitivity reaction, evident as urticarial wheals. The incoming inflammatory cells, in turn, release more proinflammatory mediators that serve to recruit and activate other cell types, thereby amplifying and extending the host response. The upregulation of inflammatory molecules, almost comparable expression of chemokines and adhesion molecules, and higher T-cell numbers in uninvolved skin is indicative of widespread immunologic activation, representing a low level priming of the cutaneous inflammatory and immunologic response apparatus, reconfirming the hypothesis of latent, minimal persistent inflammation in apparently uninvolved skin [4, 5]. This lowers the reactive threshold of mast cells to triggering stimuli and facilitates the maintenance of susceptibility to urticaria during clinical remission.

## 2. Autoimmunity and Chronic Urticaria

The autoimmune origin is the most accepted hypothesis advanced to explain inappropriate activation and degranulation of mast cells in urticaria. Immune tolerance is maintained by a balance between autoreactive lymphocytes and regulatory mechanisms that counteract them. An increase in number and/or function of naturally occurring autoreactive T-cells or diminished regulator mechanism manifests as autoimmunity. Regulatory T-cells (T(REG)), particularly the naturally occurring CD4(+) CD25(+) subset of T (REG), provide a substantial component of autoimmune counterbalance. The identification of forkhead box P3 (FOX P3) as a critical determinant of CD4(+) CD25(+) T(REG) cell development and function has provided insights into the delicate balance between autoreactive and regulatory mechanisms in autoimmune disorders including chronic autoimmune urticaria. Functional assays and phenotype analysis have revealed that T(REG) isolated from patients of autoimmune disorder exhibit reduced regulatory function as opposed to those from healthy controls. It may be concluded that reduced percentage of CD4(+) CD25(+) FOX P3(+) regulator T-cells contributes to the autoimmune pathogenic process of chronic urticaria [6]. The autoimmune pathogenic mechanism has been conceptualized on the following observations that provided the initial circumstantial evidence and impetus for further clinical and laboratory investigations that reaffirmed the concept.

- Higher prevalence of thyroid autoantibodies in chronic urticaria [7].
- A wheal-and-flare reaction on intradermal injection of autologous serum in a subpopulation of patients (positive autologous serum skin test) and reproducibility on passive transfer of serum to normal healthy control subjects [8].
- Subsequent identification of IgG antibody directed to the alpha subunit of the IgE receptor, capable of inducing positive autologous serum skin test as well as histamine release from basophils [9]. The incidence of such autoantibodies is about 30 percent and an additional 5–10 percent of patients have anti-IgE antibodies rather than anti-IgE receptor antibody [10].
- Positive association with HLA subtypes DRB*04 (DR4) and DQB 1*0302 (DQ8) [11].
- Therapeutic response to plasmapheresis [12] and intravenous immunoglobulin [13].

The evidence favouring autoimmune pathomechanism, although persuasively convincing, is incomplete. Certain issues, elucidated below, need to be addressed for unequivocal acceptance of the proposed hypothesis.

- The cutaneous response to intradermal injection of autologous serum may be due to the presence of nonimmunoglobulin vasoactive histamine releasing factors [14]. Moreover reactivity to autologous serum has been observed in subjects with allergic respiratory diseases and healthy controls [15]. ASST identifies subsets of patients exhibiting autoreactivity rather than establishing autoimmunity.
- Animal model, mandatory to establish autoimmune status of the disorder, is yet to be developed for chronic urticaria [16].
- The autoantibodies of similar specificity have been detected in sera of healthy persons and may belong to the natural repertoire. Such natural autoantibodies may become pathogenic under certain circumstances and this occurrence is dependent on the state of occupancy of the FceRI receptor by its natural ligand IgE. The urticaria results from alteration in the tissue binding of preexisting autoantibody in susceptible individuals rather than its production* de novo*. Thus the concept of conditional autoimmunity has evolved [17] in chronic urticaria.
- It has been proposed that anti-FceRI and anti-IgE autoantibodies are not actually pathogenic but are secondary to the presence of urticaria in individuals with a predisposition to develop autoimmunity [18].

The complex pathway involved in triggering, maintaining, and controlling autoantibody formation against FceRI and/or IgE remains unexplained. The autoantibodies relevant to chronic urticaria belong to complement fixing subtypes IgG1 and IgG3 [19]. The vascular leakage of such autoantibodies by local events facilitates their binding to either FceRI or IgE, cross-linking of the receptor, and complement activation that generates C5a. C5a interacts with the receptor for complement anaphylatoxin (C5a receptor) localized on the surface of mast cell MC_TC_, the subtype dominant in the skin, and participates in mast cell activation [20]. This triggers a series of intracellular events and the earliest in signal transduction involves phosphorylation of tyrosine on the beta and gamma chains of FceRI at immunoreceptor tyrosine activation motifs (ITAM). The ITAMS associate with Src-family protein tyrosine kinases (PTKs), such as Lyn and Syk, which initiate the activation of downstream effector pathways [21] and the release of preformed granule contents such as histamine, heparin, tryptase, and tumour necrosis factor-alpha (TNF-alpha), as well as the synthesis of other proinflammatory cytokines/chemokines and eicosanoids [22]. The downregulation of signal transduction and mediator release is regulated by signal regulatory proteins (SIRP) that contain immunotyrosine inhibition motifs (ITIMS) which act by recruiting SH-2 bearing tyrosine phosphatases (SHIP 1 and 2) that dephosphorylate ITAM on beta and gamma subunits of FceRI [21].

The autoantibodies to alpha chain of FceRI and/or IgE have been detected in only 35 to 40 percent and 5 to 10 percent of patients, respectively [23]. Moreover the detection of autoantibodies in the serum does not confirm their functionality and may not be always implicated in the induction of histamine release from mast cells/basophils. It is likely that other permeabilizing factors are involved in serum mediated vascular leakage.

## 3. Nonimmunologic Agonists

Nonimmunologic agonists including substance P, endorphins, enkephalins, endogenous peptides, and somatostatin may induce regulated degranulation and liberation of proinflammatory molecules from mast cells especially when the products of activated immune system lower the cutaneous mast cell release threshold [24].

## 4. Cellular Abnormalities: Basophils 

The primary abnormality in some patients of chronic urticaria might be cellular/subcellular rather than immunologically mediated autoimmune mechanism.

There is increasing evidence of altered number, structure, function, and trafficking defects in basophils. Basopenia is well documented and basophil numbers are inversely related to urticaria severity [25]. Other evidence, perhaps more convincing, is the paradoxical suppression of FceRI mediated, anti-FceRI/anti-IgE antibody induced histamine release from basophils during active disease [26]. It is still more intriguing that these basophils maintain normal response to monocyte chemotactic protein-1 (MCP-1) and bradykinin and are hyperresponsive to serum [27]. It had been reasoned that basophils in chronic autoimmune urticaria are desensitized* in vivo* to further FceRI induced activation. However, autoantibody mediated desensitization of IgE receptor seems unlikely as similar response of basophils has been observed in patients lacking autoimmune antibodies [27].

A more complex picture has emerged from insight into the dysregulated expression of molecules that are critical to signal propagation or its inhibition after IgE receptor activation [28]. Spleen tyrosine kinase (Syk) is a positive regulator of signaling through FceRI and its levels are a major determinant of basophil histamine release (HR) in normal basophils [29]. Src homology 2 (SH2) containing inositol phosphatases, SHIP-1 and SHIP-2, are negative regulators of signal propagation [30]. In chronic urticaria there is a shift in the paradigm of Syk dominated regulation of HR and unlike normal basophils altered levels of SHIP-1 and SHIP-2 correlate with the pattern of anti-IgE stimulated histamine release [31, 32].

In addition to these observations, based on the profile of* ex vivo* activation of basophils by optimal concentration of polyclonal anti-IgE, it has been confirmed that distinct basophil degranulation phenotypes exist in chronic urticaria and a bimodal stratification of basophils has been proposed [32]. Fifty percent of chronic urticaria subjects have significant reduction in basophil histamine release (HR) with anti-IgE stimulation. It is consequent to increased SHIP-2 levels and such subjects are designated anti-IgE nonresponders (CIU-NR). The remaining subjects have basophils that release more than 10 percent of histamine content after anti-IgE stimulation and are termed anti-IgE responders (CIU-R) and SHIP-1 levels in such basophils are reduced.

This pattern of basophil functional phenotypes (CIU-R and CIU-NR) appears to be independent of the existence and/or levels of autoantibodies [33] and remains stable in subjects with persistent disease. The salient features of basophil phenotypes in chronic urticaria are summarized in Table 3 [31–33].

The levels and/or expression of regulatory proteins is functional and normalizes during remission [33]. Such shift in basophil function is independent of the autoimmune status of urticaria and, in those with autoimmunity, is noted without a parallel decrease in antibody titres.

This profiling of signal proteins in basophils in conjunction with their stratification into distinct phenotypes has reaffirmed that abnormal basophil function may be a key factor in disease pathogenesis.

## 5. Mast Cells and Chronic Urticaria

A direct role of mast cells in CU is speculated (Table 4). The activating factors derived from inflammatory cell infiltrate surrounding dermal postcapillary venules [34] stimulate mast cells to secrete vasoactive molecules that activate endothelial cells. The expression of adhesion molecules is upregulated [35] and the increased vasopermeability promotes extravascular leakage of fluids and proteins leading to development of urticarial wheals.

In an* in vitro* study performed to evaluate permeabilizing activity of CU serum, a similar pattern of mast cell degranulation and increased endothelial monolayer permeability was observed after exposure of two distinct mast cell lines (LAD-2 and HMC-1) [36, 37] to CU serum. The CU serum evoked response remained unaltered after IgG depletion, reaffirming its nondependence on mast cell IgE receptor activation [38]. It was concluded that vasoactive molecules may be released from mast cells without degranulation [38].

It is likely that varied membrane receptors expressed on mast cells are selectively triggered by ligands such as IgG, peptides, microbial derivatives, and fragments of activated complement [39] and mast cells are stimulated by activating signals to synthesize vasoactive substances including lipid metabolites, cytokines, and chemokines [40, 41]. These newly formed permeabilizing factors include tumour necrosis factor-alpha (TNF-alpha), interleukin-6 (IL-6), vascular endothelial growth factor (VEGF), and platelet activating factor (PAF). These are secreted from mast cells independent of release of preformed mediators stored in granules such as histamine, serotonin, proteases, and proteoglycans [40, 41]. These permeabilizing factors facilitate the development of urticarial wheals. These observations provide an explanation for lack of correlation between detection of autoantibodies and degranulation of mast cells [42], quality and quantity of released vasoactive factors, increased vasopermeability, urticaria severity refractoriness of severe urticaria to standard first line management with antihistamines, and therapeutic response to immunosuppressive agents.

## 6. Chronic Urticaria: Immune Mediated Inflammatory Disorder

The concept of chronic urticaria being an immune mediated inflammatory disorder evolved from incidental observation that recommendation of targeted immunomodulator biologic therapy, directed at a particular cytokine/cell receptor, administered for some other inflammatory disorder ameliorated coexisting chronic urticaria [43]. It was indicative that inflammatory cascade in chronic urticaria may be triggered by altered chemokine-cytokine network and is attributed to immune dysregulation consequent to disturbed innate immunity in the disorder.

The investigation of early events of innate immunity through the study of dendritic cells that link innate and adaptive immune system has provided insights into the dysregulated immune response in chronic urticaria [44]. Plasmacytoid dendritic cells (pDC) express toll-like receptors (TLR) that are activated by natural and synthetic ligands triggering proinflammatory responses that play a key role in the pathogenesis of several inflammatory disorders including urticaria [45].

Accordingly, a study of early events of the immune response, through the activation of pDC by TLR sensing, was undertaken to evaluate immune dysregulation in chronic urticaria [44].

Plasmacytoid dendritic cells are unremarkably scattered amongst cellular infiltrate in the cutaneous lesion and their percentage in the peripheral blood mononuclear cells is unaltered and is similar to healthy controls. A normal degree of activation of pDC by the expression of costimulatory molecules and an increased constitutive STAT 1 phosphorylation on nonstimulated lymphocytes was observed. However, IFN-alpha secretion by pDC upon stimulation by CpGA was impaired and it was associated with altered IRF-7 and downregulation of TLR 9 expression, indicating a functional impairment of pDC, and was supportive of immune dysregulation in CU [44].

Several hypotheses have been proposed to explain the downregulation of TLR9 in pDC after CpGA stimulation. It is probable that IgE or anti-FceRI autoantibodies cross-link FceRI on immature pDC impairing immune function by suppressing IFN-alpha production [46]. Alternatively, regulatory receptors including BDCA-2, ILT-7, and NKp44 downmodulate IFN production [47–49]. It is also likely that histamine released into circulation from degranulating mast cells/basophils may regulate several cell types through stimulation of histamine receptors and pDC activated by CpG respond to histamine through H2 receptors with downregulation of IFN-alpha production [50].

The dysfunctional innate immune response in CU consequent to functional impairment of pDC to TLR9 activation disturbs the cytokine production by T-cells, mainly of IL-17A and IL-10 [51]. Besides, elevated serum levels of IL-1, IL-4, IL-13, IL-18, tumour necrosis factor-alpha (TNF-alpha) [52, 53], B-cell activating factor (BAFF) [54], and factors related to inflammatory process such as neopterin [55] and C-reactive protein have been documented. These observations suggest that there is an ongoing inflammatory process, creating a proinflammatory environment in chronic urticaria that in turn is responsible for an altered pattern of secretion of chemokines.

Significantly higher serum levels of chemokines (C-C and C-X-C) including CXCL8, CXCL9, CXCL10, and CCL2 have been observed in CU and are not correlated with either the clinical parameters of the disorder or the outcome of basophil histamine release (BHR) and/or ASST assays [56]. These proinflammatory chemotactic cytokines interact with chemokine receptors on the surface of inflammatory cells to trigger chemotaxis and transendothelial migration of leukocytes to the site of inflammation.

CXCL8/IL-8 is chemotactic for neutrophils, T-lymphocytes, and monocytes [56].

CXCL9/Mig, a monokine induced by interferon- (IFN-) gamma induced protein 1 (IP-10), is a type 1 C-X-C chemokine and displays strong chemoattraction for T-helper type 1 (Th 1) lymphocytes [57, 58].

C-C ligand 2 (CCL2) (monocyte chemoattractant protein 1), prototype of C-C chemokine, is secreted mainly by monocytes, as evidenced by increased mRNA expression in CD14 + cells in CU. CCL2 activates a variety of cells including monocytes, macrophages, lymphocytes, eosinophils, and basophils and is a crucial factor for the development of Th 2 responses [59].

The upregulation of chemokines in CU contributes to the maintenance of activated status of inflammatory cell subsets. Basophils in CU display an upregulation of activation/degranulation markers, CD203c and CD63, and high responsiveness to interleukin-3 (IL-3) stimulation. It is likely that activated profile of basophils is triggered by* in vivo* priming with potent basophil activating factor such as CCL2 [60].

CCL2 induces degranulation of mast cell and has a potent basophil histamine releasing activity. Furthermore, it has been documented that an assembly of circulating chemokines CCL2, CCL5, and CXCL8 play an important role in mast cell activation and generation of histamine and serotonin [61].

It may be inferred that immunologic dysregulation consequent to disturbed innate immune response alters the cytokine-chemokine network that triggers the inflammatory status contributing to the pathogenesis of CU.

## 7. Chronic Urticaria and Coagulation System

It has been observed that autologous plasma, anticoagulated with substances other than heparin, generates positive autoreactive responses in a higher percentage of patients than autologous serum skin test (ASST) [62, 63]. As serum and plasma do not differ in their autoantibody content, this observation points to a possible role of clotting factors in wheal-and-flare reaction. It has been reasoned that plasma contains more coagulation factors and complement, while consumption of such factors in serum during formation of clot is responsible for the discrepant reactivity of autologous plasma and serum. It has thus been inferred that clotting cascade may be involved in pathogenesis of urticaria [64] and this may provide an explanation for therapeutic effects noted in some patients with drugs active on coagulation system [65, 66].

The extrinsic pathway of coagulation is activated and thrombin is generated from prothrombin by activated factor X, in the presence of activated factor V and calcium ions [64].* In vitro* studies have confirmed that plasma of urticaria patients has significantly higher levels of prothrombin fragment F_1+2_, a polypeptide of 34 kDa that is released into circulation during the activation of prothrombin to thrombin by factor X [67], and severe exacerbations of urticaria are associated with a strong activation of coagulation cascade that leads to fibrin formation and fibrinolysis as shown by elevated D-dimer plasma levels.

Thrombin is a serine protease that enhances vascular permeability, activates and degranulates mast cells, and induces generation of anaphylatoxin C5a. The activation of extrinsic pathway of coagulation is thus proposed as yet another explanation.

**Figure 1:**
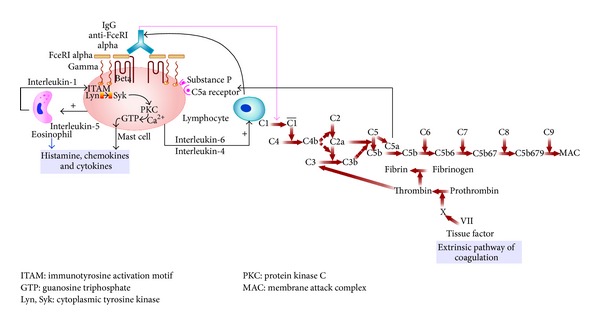
Pathogenesis of chronic urticaria: molecular intercommunication between autoimmune, complement, and coagulation cascade.

It is likely that different pathomechanistic pathways, namely, seroimmunologic autoimmune, inflammatory, cellular defects, coagulative, and complement system, are interlinked rather than separate independent cascades and there is extensive cross talk amongst them with mutual regulation of activation. They act synergistically or sequentially as either independent or interlinked pathomechanisms to activate mast cells with release of preformed mediators and/or secretion of newly synthesized vasoactive molecules to produce final clinical expression of urticaria [68, 69] (Figure 1).

The various mast cell mediators, preformed and newly synthesized, relevant to chronic urticaria, are summarized in Table 4.

Histamine is the principal vasoactive mediator and combined histamine-1 and -2 receptor responses are required for the full expression of histamine vasoactivity, including immediate vasodilation, alteration in vasopermeability, plasma extravasation, and dermal sensory nerve stimulation [69–71]. Its role in the pathogenesis of wheal is less certain and it is probable that nonhistamine mast cell mediators regulate cell recruitment. The leukotrienes, cytokines, and chemokines upregulate adhesion molecule expression on endothelial cells, promoting rolling and adhesion of leukocytes, followed by chemotaxis and transendothelial migration and cellular influx into whealing skin. These infiltrating cells in turn release proinflammatory cytokines and chemokines that serve to recruit and activate more inflammatory cells, thereby sustaining, amplifying, and extending the host response. The signals for resolution of urticaria are also not well characterized. It involves downregulation of the histamine receptor, reconstitution of integrity of the endothelial cell lining, apoptosis of the inflammatory cells, clearance of cellular debris by macrophages, and drainage of edema fluid into the vascular circulation [22].

It may be concluded that the pathogenesis of chronic urticaria is as perplexing as the disorder is intriguing and a concise pathomechanism has, as yet, not been identified that may provide a rational explanation for all cases. An incomplete understanding has hampered the search for novel, efficacious, low toxicity drugs that may be offered as alternatives in severe, unremitting urticaria, unresponsive to standard first line of management. It is, however, likely that newer insights into the complexities of pathogenesis may pave the way for evolving therapies that are more specifically tied to the pathomechanics of the disorder and provide an impetus to develop targeted immunomodulators and biological therapies that may be favorably included in therapeutic armamentarium.

## Figures and Tables

**Table 1:** Infiltrating cells: pattern in urticarial wheal, uninvolved skin, and normal healthy control subjects [4].

Cell type	Urticarial wheal	Uninvolved skin	Healthy control subjects
Mast cells	Normal count	Normal count	Normal count
Lymphocytes	Raised T-lymphocyte count	More numerous T-lymphocytes than in lesional skin	Low T-lymphocyte counts
Neutrophils	Major cellular infiltrate at 60 minutes of evolution of urticarial wheal	Significantly less infiltration than in lesional skin	Insignificant
Eosinophils	Significantly higher number	Insignificant	Insignificant
Basophil	Significant number, especially at 30 minutes of evolution, of urticarial wheal	Less but relevant number	Insignificant

**Table 2:** Cytokine, chemokine, and adhesion molecule expression: urticarial wheals, uninvolved skin, and healthy control subjects [4].

	Urticarial wheal	Uninvolved skin	Normal healthy controls
Cytokines			
Interferon gamma	High expression	Significantly low expression	Not expressed
Interleukin-4	High expression	Significantly low expression	Not expressed
Interleukin-5	High expression	Significantly low expression	Not expressed
Interleukin-8	Moderate expression	Moderate expression	Not expressed
Chemokines			
C X C R 3/CC R 3	Expression similar to control skin	High expression	Expression similar in lesional and healthy control skin
Adhesion molecules			
Cellular adhesion molecule	High expression	Intense expression	Significant expression

**Table 3:** Basophil phenotypes: profile in chronic urticaria [31–33].

Feature	Chronic urticarial (autoimmune/nonautoimmune)	
	CIU-R	CIU-NR
(1) Anti-IgE stimulation/cross-linking: HR in active disease	>10 percent of cellular content	<10 percent of cellular content
(2) Regulatory proteins	Paradigm shift	Paradigm shift
(a) Kinase	HR not determined by kinase level	HR not determined by kinase level
	Normal Syk levels	Normal Syk levels
(b) Phosphatase	Regulates HR, reduced SHIP-1 levels	Regulates HR, increased SHIP-2 levels
(3) Sensitivity to anti-IgE stimulation in remission	Heightened sensitivity	Sensitivity restored to levels as in normal healthy subjects

HR: histamine release.

CIU-R: chronic idiopathic urticaria—responders.

CIU-NR: chronic idiopathic urticaria—nonresponders.

Syk: serum tyrosine kinase.

SHIP-1: Src homology 2 (SH2) containing inositol phosphatase 1.

SHIP-2: Src homology 2 (SH2) containing inositol phosphatase 2.

**Table 4:** Mast cell mediators: relevant to chronic urticaria.

Mediator	Effect in chronic urticaria
Preformed mediators	
Histamine	Direct potent vasoactive and smooth muscle spasmogenic effects Principal mediator of vascular changes [70, 71]
Newly synthesised mediators	
Lipid mediators	
LTC4 LT B4 PGD2	Actions similar to histamine Potentiate vasodilatation, vascular permeability, and smooth muscle contraction Chemotactic for neutrophils and eosinophils [72]
Cytokines and chemokines	
Tumour necrosis factor-alpha	Newly synthesised as well as preformed
	Upregulates expression of adhesion molecules on endothelial cells Promotes leukocyte rolling and adhesion Chemotactic for neutrophils [73]
Interleukin-1	Proinflammatory cytokine Lymphocyte activator [74] Activates mast cells after release from leukocyte [75]
Interleukin-4	Chemotactic for neutrophils Recruits eosinophils [76]
Interleukin-5	Recruits eosinophils [77]
Interleukin-6	Proinflammatory cytokine Activates lymphocytes [78]
Interleukin-8/CXCL2	Member of C X C chemokines Potent neutrophil chemoattractant Involved in neutrophil degranulation, respiratory burst, and adhesion to endothelial cells [79]
MCP-1/CCL2	Chemoattractant for eosinophils
MIP-1 alpha/CCL3	Chemoattractant for eosinophils
Interleukin-16	Chemoattractant for T-lymphocytes
RANTES/CCL5	Chemoattractant for eosinophils [79]

LTC4: leukotriene C4, LTB4: leukotriene B4, PGD2: prostaglandin D2, MCP-1: monocyte chemotactic protein-1, MIP-1 alpha: monocyte inflammatory peptide-1, RANTES: regulated upon activation normal T-cell expressed and secreted.
